# Convective heat and mass transfer rate on 3D Williamson nanofluid flow via linear stretching sheet with thermal radiation and heat absorption

**DOI:** 10.1038/s41598-023-36836-4

**Published:** 2023-06-19

**Authors:** Shiva Jagadeesh, Marpadaga Chenna Krishna Reddy, Nainaru Tarakaramu, Hijaz Ahmad, Sameh Askar, Sherzod Shukhratovich Abdullaev

**Affiliations:** 1 Department of Mathematics, Osmania University, Hyderabad, Telangana, 500007 India; 2 Department of Mathematics, Basic Sciences and Humanities, Mohan Babu University, Sree Sainath Nagar, Tirupati, A.P 517102 India; 3 Department of Mathematics, Basic Sciences and Humanities, Sree Vidyanikethan Engineering College, Sree Sainath Nagar, Tirupati, A.P 517102 India; 4 Section of Methamatics, International Telematic University Uninettuno, Corso Vittorio Emanuele II, 3900186 Roma, Italy; 5 Department of Statistics and Operations Research, College of Science, King Saud University, Riyadh, 11451 Saudi Arabia; 6 Senior Researcher, Faculty of Chemical Engineering, New Uzbekistan University, Tashkent, Uzbekistan; 7 Senior Research, Department of Science and Innovation, Tashkent State Pedagogical University Named After Nizami, Tashkent, Uzbekistan

**Keywords:** Engineering, Mathematics and computing

## Abstract

A mathematical analysis is communicated to the thermal radiation and heat absorption effects on 3D MHD Williamson nanoliquid (NFs) motion via stretching sheet. The convective heat and mass boundary conditions are taken in sheet when liquid is motion. As a novelty, the effects of thermal radiation, heat absorption and heat and mass convection are incorporated. The aim is to develop heat transfer. Williamson NFs are most important source of heat absorption, it having many significant applications in “energy generation, HT, aircraft, missiles, electronic cooling systems, gas turbines” etc. The suitable similarity transformations have been utilized for reduce basic governing P.D. E’s into coupled nonlinear system of O.D. E’s. Obtained O.D. Es are calculated by help of R–K–F (“Runge–Kutta–Fehlberg”)4th order procedure with shooting technique in MATLAB programming. We noticed that, the skin friction coefficient is more effective in Williamson liquid motion when compared with NFs motion with higher numerical values of stretching ratio parameter, Williamson liquid motion is high when compared to NFs motion for large values of magnetic field. We compared with present results into previous results for various conditions. Finally, in the present result is good invention of previous results.

## Introduction

New scientists have been tremendous interest for doing research in non-Newtonian liquid. Due to their broad real applications in engineering fields (“such as biological sciences, geophysics, and chemical and petroleum industries, food processing, performance of lubricants, plastic manufacture, movement of biological fluids, polymer processing, ice and magma motion”) . Property-wise non-Newtonian liquid models are “Powell-Eyring fluid, Sisko, Jeffrey fluids, Prandtl fluid, Casson fluid, and Williamson fluid models”. Out of these fluid models Williamson liquid model is attractive area for new generation. It describes the motion of shear thinning non-Newtonian liquid. Williamson fluid (“motion via stretching surface applications is copper spiralling, cod depiction, warm progressing, extrusion, and melting of high molecular weight polymers”) model is considered minimum $\mu_{\infty }^{*}$ and maximum viscosities $\mu_{0}^{*}$. Williamson^1^ developed the motion of pseudoplastic liquid and results are verified experimentally. Last few years, some of scientists^2,3^ develop Williamson liquid motion on 2D surface. Khan et al.^4^ presented Williamson nanoliquid motion via oscillating SS. The Williamson NFs via SS with variable viscosity was explored by Khan et al.^5^. Hashim et al.^6^ presents the Thermophysical features of non-Newtonian liquid motion with variable thermal conductivity. The stagnation point (SP) motion of Williamson liquid via SS was explored^7,8^. Some of investigators^9,10^ discussed the peristaltic motion of Williamson liquid. Rehman et al.^11^ developed numerical analysis of dual convection. The heat transfer of Williamson liquid motion via stretching cylindrical surface was explored^12,13^. Aziz et al.^14^ studied convective heat transport and volumetric entropy generation in Powell-Eyring hybrid NFs via SS. Hussain and Jamshed^15^ examined hybrid NFs flowing properties and thermal transport via slippy surface. Bilal et al.^16^ exhibited Williamson liquid motion via cylindrical surface by using Keller-Box method. Recently, some of scientists^17–19^ presented 3D liquid motion via SS. Also, some of authors^20–22^ described numerical solutions of non-Newtonian liquid motion via sheet. Some of Scientists^23–25^ explored non-Newtonian liquid motion via SS. Mishra et al.^26^ discussed MHD motion of power-law liquid on SS with non-uniform heat source. Jamshed et al.^27^ presented entropy in porous medium of Williamson NFs motion via exponentially horizontal plate. The analytical results of couple stress fluid motion via permeable sphere was created Aparna et al.^28^. Venkateswarlu et al.^29^ explored the dissipative motion of propylene-glycol and water mixture-based hybrid nanoliquid via sphere. Jamshed et al.^30,31^ developed Williamson NFs motion via SS.

The MHD reflects dynamic activities via a stretching surface (SS). This liquid motion is electrically conducting, it established with magnetic field. The electrically conducting and heat transfer (HT) motion has many applications (“configuration orientation regarding structure of boundary layer, energy extractions in geothermal field, MHD accelerators and power generators, fluid droplets sprays and flow meters, electrostatic precipitation, polymer technology, centrifugal separation of matter from fluid, petroleum industry, magnetohydrodynamic generators, cooling systems, metallurgy, nuclear reactors, crystal growth, fluid metals and aerodynamics, accelerators, pumps, solar physics, plasma confinement, cosmology ext.”) in modern industry and engineering fields, science technology. The introduction of Magnetohydrodynamics (MHD) was established by Roberts^32^. Jamil and Haleem^33^ obtained unsteady motion of fractionalized magnetohydrodynamic Jeffrey liquid via porous plate with linear slip effect. The natural convection motion of non-Newtonian liquid via different surface was focused^34–36^. The convection motion via various sheets was explored^37–39^. The MHD motion of Erying-Powell liquid via SS was discussed^40–42^. The clearance between ceramic outer ring and steel pedestal on sound radiation was discussed^43^. Some of authors^44–46^ presented Eyring-Powell liquid via SS. He et al.^47^ presented microwave imaging of 3D dielectric-magnetic penetrable objects. Tamoor et al.^48^ exhibited the MHD Casson liquid motion induced by stretched cylinder. MHD peristaltic transport of NFs (“copper–water”) in artery with mild stenosis for different shapes of nanoparticles is studied Devaki et al.^49^. Mahabaleshwar et al.^50^ examined the MHD Couple stress liquid due to perforated sheet via linear stretching with radiation. The MHD motion via SS was presented by^51–53^. Recently, some of investigators^54–56^ developed suction or injection for gravity modulation mixed convection in micropolar liquid via inclined sheet. The 3D MHD non-Newtonian NFs via SS was exhibited^57–60^. Some of the interesting and related research was studied by^61–63^. Recently^64–66^, developed MHD and convective heat transfer motion via SS. The non-Newtonian liquid motion was studied^67–69^.

The impact of thermal radiative (TR) motion has normally known as variance b/w ambient energy and thermal energy. Which is used in several fields (“biomedicine, space machinery, drilling process, cancer treatment, high temperature methods, and power generation etc. Also, several industrial processes, include nuclear reactors, power plants, gas turbines, satellites, missiles technology etc.”) of technologies. Satya Narayana et al.^70^ focussed the thermal radiation effect on unsteady motion via SS. Kandasamy et al.^71^ analysed thermal and Solutal effect on heat ad mass transfer induced due to a NFs via porous vertical plate. The mixed convection and TR on non-aligned Casson liquid via SS was discussed Mehmood et al.^72^ exhibited the radiative motion on 2D Casson liquid past a moving wedge. Masthanaiah et al.^73^ presented heat generation on cold liquid with viscous dissipation via parallel plates. The NFs motion via radiative sheet was examined^74,75^. Recently, some of related, interacted and motivated work presented^76–78^.

The current work numerical analysis, which is enables the young researchers to compute of convective heat and mass transfer on 3D Williamson NFs motion via SS. The effect of thermal radiation, heat absorption, MHD, convective heat and mass transfer are considered in this study. It has several applications in industrial processes, petroleum industry, nuclear reactors, power plants, molecular weight polymers, energy extractions in geothermal field, power generators, polymer technology, magnetohydrodynamic generators, cooling systems, nuclear reactors, crystal growth, biomedicine, space machinery, drilling process, cancer treatment, etc.

The main motivations of current work are:

(a) The convective heat and mass transfer boundary conditions on Williamson NFs motion, (b). The effect of heat absorption and thermal radiation is enhancement of heat transportation in Williamson NFs via SS.

(c) Particularly, numerical values of “Thermal Radiation”, “Magnetic field”, “Lewis number and Prandtl numbers” leads to minimum heat and mass transfer rate are obtained.

The present results are justified through comparison by previous study as shown Table 1 and Table 2.

**Table 1:** Comparison of Initial values in the absence of $\lambda = 0$ and $\alpha = 0$.

$\alpha$	Wang ^79^$- f^{\prime\prime}\left( 0 \right)$	Ariel (Exact solution) ^80^$- f^{\prime\prime}\left( 0 \right)$	Present study $- f^{\prime\prime}\left( 0 \right)$	Wang ^79^$- g^{\prime\prime}\left( 0 \right)$	Ariel (Exact solution) ^80^$- g^{\prime\prime}\left( 0 \right)$	Present study $- g^{\prime\prime}\left( 0 \right)$
0.00	1.000000		1.00000	0.0000		0.000000
0.10			1.02026			0.066847
0.20		1.039511	1.03949		1.148745	0.148737
0.25	1.048813		1.04881	0.194564		0.194564
0.30			1.05795			0.243364
0.40		1.075795	1.07578		1.349214	0.349210
0.50	1.093097		1.09309	0.465205		0.465206
0.60		1.109951	1.10994		1.590532	0.590530
0.70			1.12639			0.724533
0.75	1.134485		1.13448	0.794622		0.794627
0.80		1.142491	1.14248		0.866685	0.866684
0.90			1.15825			1.016539
1.00	1.173720	1.173722	1.17372	1.173720	1.173722	1.173720

**Table 2:** Comparison of final values in the absence of $\lambda = 0$ and $\alpha = 0$.

$\alpha$	Wang ^79^$f\left( \infty \right)$	Present study $f\left( \infty \right)$	Wang ^80^$g\left( \infty \right)$	Present study $g\left( \infty \right)$
0.00	1.000000	1.000000	0.000000	0.000000
0.10		0.957644		0.114869
0.20		0.922653		0.232361
0.25	0.907075	0.907075	0.257986	0.257986
0.30		0.892531		0.300367
0.40		0.866033		0.379226
0.50	0.842360	0.842360	0.451671	0.451671
0.60		0.820962		0.518960
0.70		0.801441		0.581991
0.75	0.792308	0.792308	0.612049	0.612126
0.80		0.783500		0.641433
0.90		0.766909		0.697797
1.00	0.751527	0.751527	0.751527	0.751485

(d) In future we can developed 3D non-Newtonian NFs motion via permeable stretching sheet by computational analysis.

### Mathematical formulation

**Figure 1:**
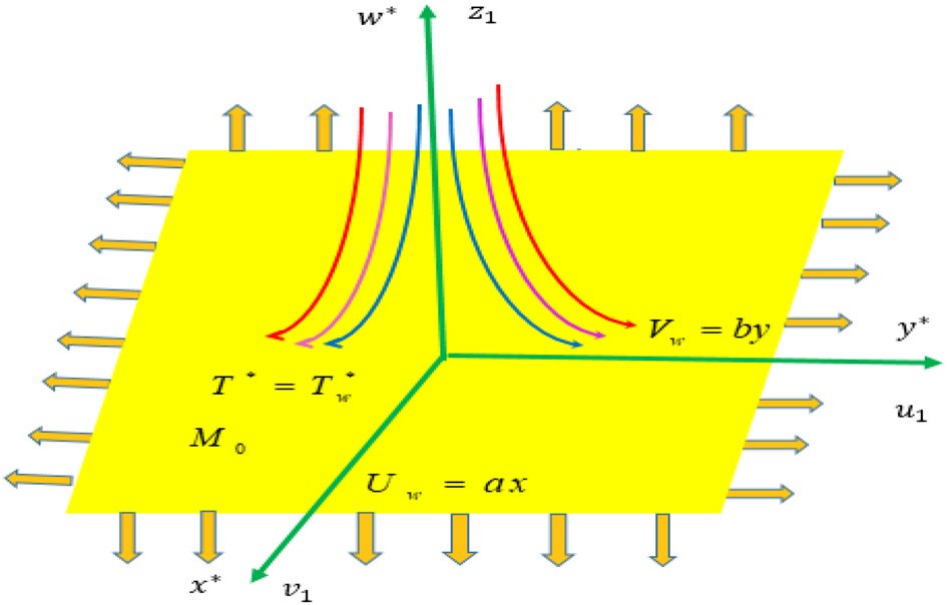
Physical model of the Problem.

Convective heat and mass transfer on 3D magnetohydrodynamic Williamson nanoliquid motion via linear stretching surface with chemical reaction is consider. Which is assumed that stretching along $x^{*} ,\,y^{*}$-surface, the fluid flow direction along $z^{*} > 0$ and flow is induced by a stretching at $z^{*} = 0$ as displayed in Fig. 1. The non-uniform magnetic field $M_{0}$ is taken in liquid motion direction. The stretching velocities along $x^{*} ,\,y^{*}$-directions as $U_{w}^{*} = a_{1} x^{*}$ and $V_{w}^{*} = b_{1} y^{*}$ is considered, respectively. The general equations of the Williamson liquid motion for conservative of mass, conservative of momentum is given below:

1 $$\nabla .V^{*} = 0,$$

2 $$\rho^{*} \frac{{dV^{*} }}{{dt^{*} }} = divS^{*} + \rho^{*} b_{1} ,$$

Williamson liquid model Equations are given by Ref.^12,15,81,82^:

3 $$S^{*} = p^{*} I_{1} + \tau^{*} ,$$

4 $$\tau^{*} = \left[ {\left( {\mu_{0}^{*} - \mu_{\infty }^{*} } \right)\left( {1 - \Gamma^{*} \dot{\gamma }^{*} } \right)^{ - 1} } \right]A_{1} ,$$

The “first Rivlin-Erickson tensor” $A_{1}$ and shear rate $\dot{\gamma }^{*}$ is defined as below:

$$A_{1} = \left( {\nabla \times V} \right) + \left( {\nabla \times V} \right)^{T}$$

$$\dot{\gamma }^{*} = \frac{1}{\sqrt 2 }\sqrt {\sum\limits_{i} {\sum\limits_{j} {\dot{\gamma }_{ij}^{*} \dot{\gamma }_{ij}^{*} } } \pi^{*} } = \frac{{\sqrt {\pi^{*} } }}{\sqrt 2 },$$

$$\pi^{*} = trace(A_{1}^{2} )$$

Consider $\mu_{\infty }^{*} = 0$ and $\Gamma^{*} \dot{\gamma }^{*} < 1$ thus Eq. (4) can be expressed as

5 $$\tau^{*} = \left[ {\mu_{0}^{*} \left( {1 - \Gamma^{*} \dot{\gamma }^{*} } \right)} \right]A_{1} ,$$

Under consideration of above, the governing equations of conservative of mass, conservative of momentum, conservative of energy and concentration are formed as following Ref.^12^:

6 $$\nabla \cdot \vec{V} = 0$$

7 $$\vec{V}\cdot \nabla u_{1} = \upsilon^{*} \frac{{\partial^{2} u_{1} }}{{\partial \left( {z^{*} } \right)^{2} }} + \sqrt {2\upsilon^{{^{*} }} } \,\Gamma^{*} \frac{{\partial u_{1} }}{{\partial z^{*} }}\frac{{\partial^{2} u_{1} }}{{\partial \left( {z^{*} } \right)^{2} }} - \frac{{\sigma^{*} M_{0}^{2} }}{{\rho^{*} }}u_{1}$$

8 $$\vec{V}\cdot \nabla v_{1} = \upsilon^{*} \frac{{\partial^{2} v_{1} }}{{\partial \left( {z^{*} } \right)^{2} }} + \sqrt {2\upsilon^{{^{*} }} } \,\Gamma^{*} \frac{{\partial v_{1} }}{{\partial z^{*} }}\frac{{\partial^{2} v_{1} }}{{\partial \left( {z^{*} } \right)^{2} }} - \frac{{\sigma^{*} M_{0}^{2} }}{{\rho^{*} }}v_{1}$$

9 $$\left. \begin{aligned} \vec{V}\cdot \nabla T^{*} = & \alpha^{*} \frac{{\partial^{2} T^{*} }}{{\partial \left( {z^{*} } \right)^{2} }} + \tau_{1} \left( {D_{B} \frac{{\partial C^{*} }}{{\partial z^{*} }}\frac{{\partial T^{*} }}{{\partial z^{*} }} + \frac{{D_{{T^{*} }} }}{{T_{\infty }^{*} }}\left( {\frac{{\partial T^{*} }}{{\partial z^{*} }}} \right)^{2} } \right) - \frac{1}{{\left( {\rho^{*} C_{p}^{*} } \right)_{f} }}\frac{{\partial q_{r} }}{\partial z} \\ & - \frac{{Q_{0} \left( {T^{*} - T_{\infty }^{*} } \right)}}{{\left( {\rho^{*} C_{p}^{*} } \right)_{f} }} \\ \end{aligned} \right\}$$

10 $$\vec{V}\cdot \nabla C^{*} = D_{B} \frac{{\partial^{2} C^{*} }}{{\partial \left( {z^{*} } \right)^{2} }} + \frac{{D_{{T^{*} }} }}{{T_{\infty }^{*} }}\left( {\frac{{\partial^{2} T^{*} }}{{\partial \left( {z^{*} } \right)^{2} }}} \right)$$

The boundary conditions of the present model are

11 $$\begin{gathered} as\,\,\,\,\,z^{*} = 0\,\,\,\,\,as\,\,\,\,u_{1} = a_{1} x^{*} \,\,\,\,\,\,\,v_{1} = b_{1} y^{*} \,\,\,\,\,\,\,\,\,w^{*} = 0,\,\,\,\,\,\, - k^{*} \frac{{\partial T^{*} }}{{\partial z^{*} }} = h_{1}^{*} (T_{f}^{*} - T^{*} )\,\,\,\,\,\, - D\left( {\frac{{\partial C^{*} }}{{\partial z^{*} }}} \right) = h_{2}^{*} \left( {C_{f}^{*} - C^{*} } \right) \hfill \\ at\,\,\,\,\,z^{*} \to \infty \,\,\,\,u_{1} \to 0\,\,\,\,\,\,\,v_{1} \to 0,\,\,\,\,\,\,\,\,\,\,T^{*} \to T_{\infty }^{*} ,\,\,\,\,\,\,C^{*} \to C_{\infty }^{*} \hfill \\ \end{gathered}$$

The radiative heat flux $q_{r}$ which is given by Quinn Brewster^83^ is given by

12 $$q_{r} = - \frac{{4\sigma^{*} }}{{3k^{*} }}\frac{{\partial T^{*4} }}{{\partial z^{*} }},$$

Neglected higher order terms we get

13 $$\left( {T^{*} } \right)^{4} = 4T^{*} \left( {T_{\infty }^{*} } \right)^{3} - \left( {T_{\infty }^{*} } \right)^{4}$$

Differentiate above heat flux equation, we get

14 $$\frac{{\partial q_{r} }}{{\partial z^{*} }} = - \frac{{16\sigma^{*} T_{\infty }^{3} }}{{3k^{*} }}\frac{{\partial T^{*} }}{{\partial z^{*} }}$$

Substituting Eq. (14) in Eq. (4), we get below Expression

15 $$\left. \begin{aligned} \vec{V}\cdot \nabla T^{*} = &\, \alpha_{m}^{*} \frac{{\partial^{2} T^{*} }}{{\partial \left( {z^{*} } \right)^{2} }} + \frac{1}{{(\rho^{*} C^{*} )_{f} }}\left( {\frac{{16\sigma^{*} \left( {T_{\infty }^{*} } \right)^{3} }}{{3k^{*} }}\frac{{\partial^{2} T^{*} }}{{\partial \left( {z^{*} } \right)^{2} }}} \right) \\ & + \frac{{(\rho^{*} C_{p}^{*} )}}{{(\rho^{*} C_{p}^{*} )_{f} }}\left( {D_{B} \frac{{\partial T^{*} }}{{\partial z^{*} }}\frac{{\partial C^{*} }}{{\partial z^{*} }} + \frac{{D_{T} }}{{T_{\infty }^{*} }}\left( {\frac{{\partial T^{*} }}{{\partial z^{*} }}} \right)^{2} } \right) - \frac{{Q_{0} (T^{*} - T_{\infty }^{*} )}}{{(\rho^{*} C_{p}^{*} )_{f} }} \\ \end{aligned} \right\}$$

The similarity transformations as below

16 $$\left. \begin{gathered} \eta = \sqrt {\frac{{a_{1} }}{{\upsilon^{*} }}} z^{*} ,\,\,\,\,\,\,\,\,u_{1} = a_{1} x^{*} f^{\prime}(\eta ),\,\,\,\,\,\,\,\,v_{1} = a_{1} y^{*} g^{\prime}(\eta ),\,\,\,\,\,\,\,\,w_{1} = - \sqrt {a_{1} \upsilon^{*} } (f(\eta ) + g(\eta )) \hfill \\ \,\,\,\,\,\,\,\,\,\,\,\,\,\,\,\,\,\,\,\,\,\,\,\,\,\,\,\,\,\,\theta (\eta ) = \frac{{T^{*} - T_{\infty }^{*} }}{{T_{w}^{*} - T_{\infty }^{*} }},\,\,\,\,\,\,\,\,\phi (\eta ) = \frac{{C^{*} - C_{\infty }^{*} }}{{C_{w}^{*} - C_{\infty }^{*} }} \hfill \\ \end{gathered} \right\}$$

Using above Eq. (16), we are converting Eq. (7), (8), (9), (10) and (15) into below format

17 $$f^{\prime\prime\prime}\left( {1 + \lambda f^{\prime\prime}} \right) - (f^{\prime})^{2} + f^{\prime\prime}(f + g) - Mf^{\prime} = 0$$

18 $$g^{\prime\prime\prime}\left( {1 + \lambda g^{\prime\prime}} \right) - (g^{\prime})^{2} + g^{\prime\prime}(f + g) - Mg^{\prime} = 0$$

19 $$\theta^{\prime\prime}(1 + R_{d} ) + \Pr \left( {(f + g)\theta^{\prime} + N_{b} \theta^{\prime}\phi^{\prime} + N_{t} \left( {\theta^{\prime}} \right)^{2} + H\theta } \right) = 0$$

20 $$\phi^{\prime\prime} + \Pr Le\left( {f + g} \right)\phi^{\prime} - \left( {{\raise0.7ex\hbox{${N_{t} }$} \!\mathord{\left/ {\vphantom {{N_{t} } {N_{b} }}}\right.\kern-0pt} \!\lower0.7ex\hbox{${N_{b} }$}}} \right)\theta^{\prime\prime} = 0$$

Corr. BC’s as below

21 $$\left. \begin{gathered} at\,\,\eta = 0\,\,\,\,f = 0,\,\,\,\,\,\,\,g = 0,\,\,\,\,\,\,\,\,f^{\prime} = 1,\,\,\,\,\,\,\,g^{\prime} = \alpha ,\,\,\,\,\,\,\theta ^{\prime} = - \Gamma_{1} (1 - \theta ),\,\,\,\,\phi ^{\prime} = - \Gamma_{2} (1 - \phi ) \hfill \\ as\,\,\,\eta \to \infty \,\,\,f^{\prime} \to 0,\,\,\,\,g^{\prime} \to 0,\,\,\,\,\,\theta \to 0,\,\,\,\,\,\phi \to 0 \hfill \\ \end{gathered} \right\}$$

The skin-friction coefficient and local Nusselt number are

22 $$\begin{gathered} C_{fx}^{*} = \frac{{\tau_{wx} }}{{\rho^{*} u_{w}^{2} }},\,\,\,C_{fy}^{*} = \frac{{\tau_{wx} }}{{\rho^{*} v_{w}^{2} }},\,\,\,\,Nu_{x} = \frac{{x^{*} q_{w} }}{{k^{*} (T_{f}^{*} - T_{\infty }^{*} )}}, \hfill \\ where \hfill \\ \tau_{w} = \frac{{\partial u_{1} }}{{\partial z^{*} }} + \frac{{\Gamma^{*} }}{\sqrt 2 }\left( {\frac{{\partial u_{1} }}{{\partial z^{*} }}} \right)^{2} ,\,\,\,\,\,\tau_{w} = \frac{{\partial v_{1} }}{{\partial z^{*} }} + \frac{{\Gamma^{*} }}{\sqrt 2 }\left( {\frac{{\partial v_{1} }}{{\partial z^{*} }}} \right)^{2} \hfill \\ \end{gathered}$$

While dimensionless forms of Skin friction coefficient and Nusselt number are given below:

23 $$\left. \begin{gathered} {\text{Re}}_{x}^{1/2} C_{fx} \,\,\,\,\, = f^{\prime\prime}(0) + \frac{\lambda }{2}f^{v} (0),\,\,\,\,\,{\text{Re}}_{x}^{1/2} C_{fy} \,\,\,\,\, = g^{\prime\prime}(0) + \frac{\lambda }{2}g^{v} (0) \hfill \\ {\text{Re}}_{x}^{ - 1/2} Nu_{x} = - \left( {1 + R_{d} } \right)\theta ^{\prime}(0),\,\,\,\,\,ShRe_{x}^{ - 1/2} \,\,\,\,\,\,\,\, = - \phi ^{\prime}(0) \hfill \\ \end{gathered} \right\}$$

## Results and Discussions:

**Figure 2:**
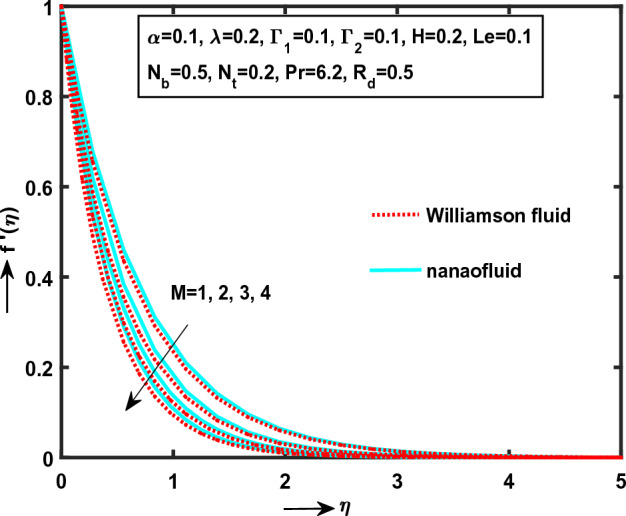
Impact of $M$ on $f^{\prime}(\eta )$.

**Figure 3:**
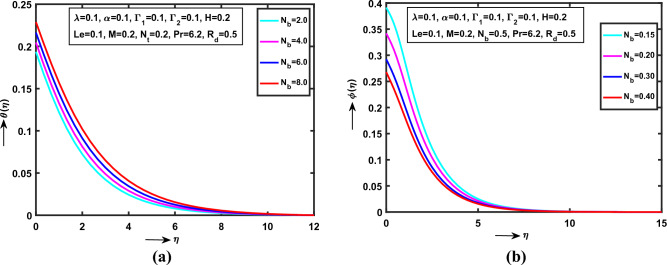
(**a**) Impact of $N_{b}$ on $\theta (\eta )$, (**b**) Impact of $N_{b}$ on $\phi (\eta )$.

**Figure 4:**
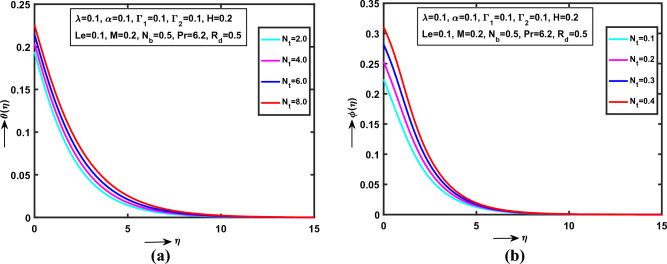
(**a**) Impact of $N_{t}$ on $\theta (\eta )$, (**b**) Impact of $N_{t}$ on $\phi (\eta )$.

**Figure 5:**
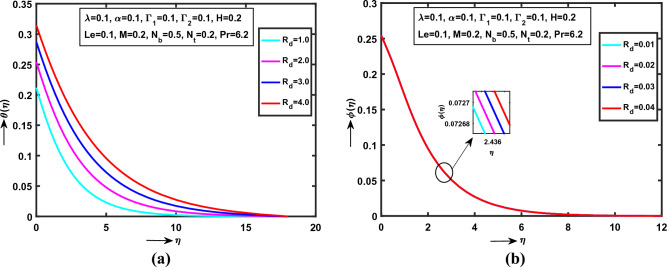
(**a**) Impact of $R_{d}$ on $\theta (\eta )$, (**b**) Impact of $R_{d}$ on $\phi (\eta )$.

**Figure 6:**
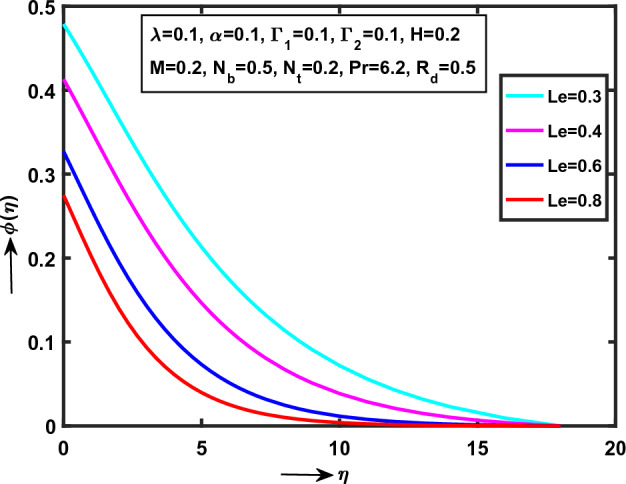
Impact of $Le$ on $\phi (\eta )$.

**Figure 7:**
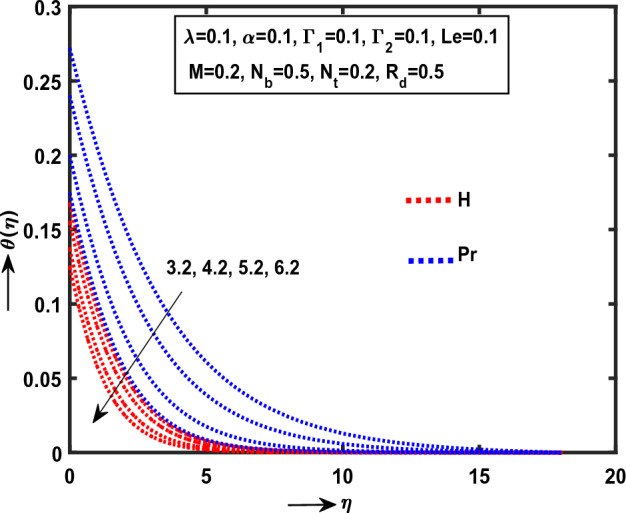
Impact of $H$, $\Pr$ on $\theta (\eta )$.

**Figure 8:**
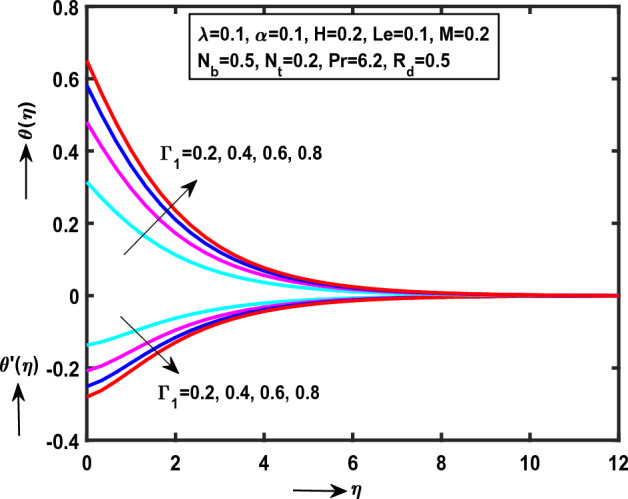
Impact of $\Gamma_{1}$ on $\theta (\eta )$,$\theta ^{\prime}(\eta )$.

**Figure 9:**
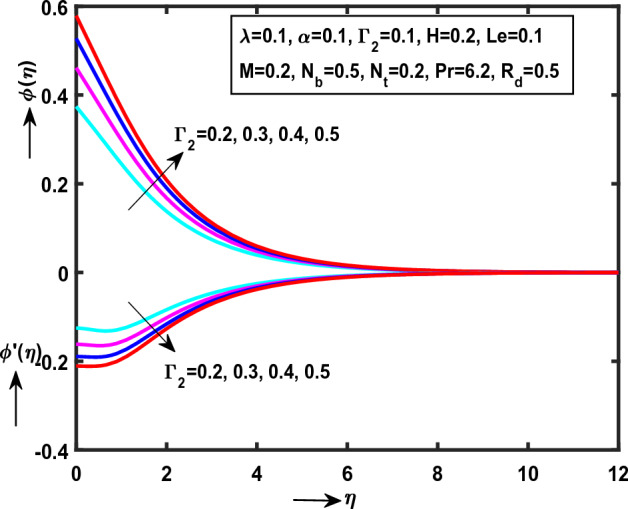
Impact of $\Gamma_{2}$ on $\phi (\eta )$,$\phi ^{\prime}(\eta )$.

**Figure 10:**
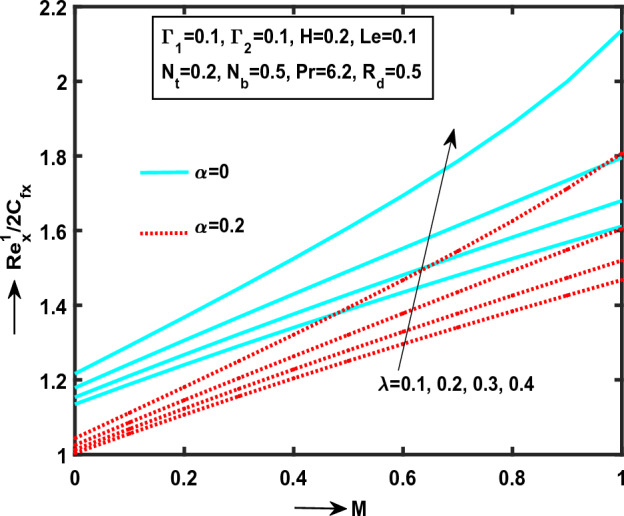
Impact of $\lambda$ on ${\text{Re}}_{x}^{1/2} C_{fx}$.

**Figure 11:**
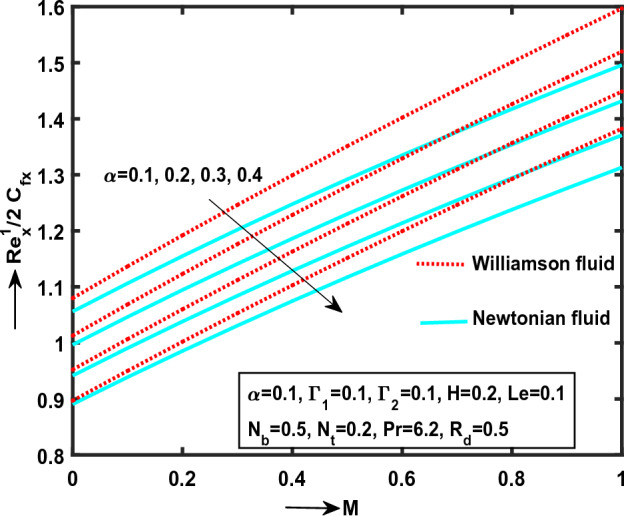
Impact of $\alpha$ on ${\text{Re}}_{x}^{1/2} C_{fx}$.

The transformed Eqs. (17), (18), (19) and (20) with B. C’s Eq. (21) has been solved numerically by Runge–Kutta–Fehlberg (R–K–F) 4th order algorithm along with shooting procedure. To develop the outstanding results of velocities $\left( {f^{\prime}(\eta ),\,\,g^{\prime}(\eta )} \right)$ flows along axial and transverse directions, heat transfer $\left( {{\text{Re}}_{x}^{ - 1/2} Nu_{x} } \right)$ rate due to related physical parameters involved in this investigation with numerical solutions are described through their plotted graphs: Figs. 2, 3, 4, 5, 6, 7, 8, 9, 10, 11, and displays tabulate values of skin friction coefficients in $x^{*} ,\,\,y^{*}$-directions. Throughout the study is discussed different Williamson fluid case and Newtonian case and the Prandtl number $\left( {\Pr = 6.2} \right)$ value of water is taken in this study.

Figure 2 demonstrate that, the significant effect of $M$(“Magnetic field Parameter”) on $f^{\prime}(\eta )$ (“axial direction”) in the presence of Williamson and nanoliquid motion behaviour with particular enlarge scientific values of $M$. It is noticed, the curvature monotonically smoothly down at the point $\eta = 0.01136$ (approximate value). Moreover, the Williamson fluid motion curve is near to the convergent area comparing nanofluid motion curvature. Finally, we decide the Williamson fluid is more significant flow more than the nanofluid flow. Physically, $M$ is proportional to (“electrical conductivity”)$\sigma$ and the magnetic field variation of $M$ leads to the Lorentz force. The Lorentz force yields high resistance to transfer phenomena.

Figure 3a,b analysed that the $N_{b}$(“Brownian motion parameter”) on $\theta (\eta )$, $\phi (\eta )$ respectively. It is observed, the $\theta (\eta )$ strictly raises with various statistical values of $N_{b}$. Also, noticed that, the curve is monotonically increases between the region $0 < \eta \le 0.0\,0\,3\,2\,7\,6$ and the convergent point at $\eta = 0.0\,0\,3\,2\,7\,6$ while reverse trend shows $\phi (\eta )$ profile for large enlarge values of $N_{b}$. The concentration curve converges point at $\eta = 0.0\,0\,3\,1\,7\,6$. Physically, $N_{b}$ is interrelated to the $D_{B}$ (“Brownian diffusion”) and $\upsilon^{*}$ (“Kinematic viscosity”). Due to, low kinematic viscosity in motion of Williamson NFs released high temperature on SS.

Figure 4a,b predicts that the $N_{t}$ (“Thermophoresis Parameter”) on $\theta (\eta )$,$\phi (\eta )$ respectively in Williamson nanofluid flow. It is detected that the both $\theta (\eta )$, $\phi (\eta )$ monotonically increases with $\lambda = \alpha = \Gamma_{1} = \Gamma_{2} = Le = 0.1$, $H = M = 0.2$, $R_{d} = 0.5$ and $\Pr = 6.2$. Also, recognized that the both heat and concentration curves is strictly increases between the area $0 < \eta \le 0.0\,0\,5\,0\,8\,1$ and the exact convergent point at $\eta = 0.0\,0\,5\,0\,8\,1$. Physically, $N_{t}$ is proportional to thermal diffusion $D_{T}$. The high thermal diffusivity produces more temperature and concentration of Williamson NFs motion via SS.

Figures 5a,b illustrate that the $R_{d}$ (“Thermal Radiation parameter”) in Williamson nanofluid flow on $\theta (\eta )$, $\phi (\eta )$ respectively. It is observed that the both $\theta (\eta )$, $\phi (\eta )$ monotonically increases with $\lambda = \alpha = \Gamma_{1} = \Gamma_{2} = Le = 0.1$, $H = M = N_{t} = 0.2$, $N_{b} = 0.5$ and $\Pr = 6.2$. Also, noticed that the both heat and concentration curves is strictly increases within the region $0 < \eta \le 0.0\,0\,5\,0\,5\,2$ and the exact convergent point at $\eta = 0.0\,0\,5\,0\,5\,2$. Physically, $R_{d}$ is inversely proportional to mean absorption coefficient.

Figure 6 presented that the $Le$ (“Lewis number”) on $\phi (\eta )$. It is clear that the concentration curve smoothly down in Williamson NFs fluid flow with various physical parameter values of $\lambda = \alpha = \Gamma_{1} = \Gamma_{2} = 0.1$, $H = M = N_{t} = 0.2$, $\Pr = 6.2$ and $R_{d} = N_{b} = 0.5$. Moreover, the curve monotonically decreases within the region $0 < \eta \le 0.0\,0\,3\,9\,0\,5$ and convergent area at $\eta = 0.0\,0\,3\,9\,0\,5$ approaches to zero. Physically, $Le$ is ratio between thermal conductivity $\alpha_{m}$ and Brownian diffusion $D_{B}$. The high Brownian diffusion released low concentration in Williamson NFs motion via SS.

Figure 7 explained that the both $\Pr$(“Prandtl number”), $H$ (“Heat source Parameter”) on $\theta (\eta )$. It is clear that the energy layer smoothly down in Williamson NFs liquid motion for fixed enlarge values of respected physical parameters. Moreover, it is observed that the Prandtl curve converges monotonically within the region $0 < \eta \le 0.0\,0\,1\,2\,7\,1$ at $\theta = 8.5$ and consequently the heat absorption curvature monotonically down between regions $0 < \eta \le 0.0\,0\,1\,9\,4$ at $\theta = 8.5$. Physical,$\Pr$,$H$ are inversely proportional to the thermal conductivity, fluid density respectively.

Figure 8 demonstrate the impact of $\Gamma_{1}$(“Thermal Biot number”) monotonically increases on $\theta (\eta )$ while reverse trend on $\theta ^{\prime}(\eta )$. It is clear that, $\theta (\eta )$ and heat transfer rate are opposite behaviour for enlarge values of $\Gamma_{1}$. Physically, the thermal Biot number is inversely proportional to thermal conductivity $k^{*}$. While the behaviour follows impact of $\Gamma_{2}$(“Concentration Biot number”) on $\phi (\eta )$, $\phi ^{\prime}(\eta )$ as presented in Fig. 9. Physically, $\Gamma_{2}$ is proportional to wall mass transfer $k_{m}^{*}$.

Figure 10 determined that the $\lambda$ (“Williamson parameter”) on ${\text{Re}}_{x}^{1/2} C_{fx}$ with presence and absence of Williamson liquid. It is observed that the skin friction coefficient strictly raises along $x^{*}$-directions for ascending values of $\lambda$. Moreover, in the presence and absence of Williamson fluid against $M$ along $x^{*}$ direction. Comparing the presence of $\alpha$ in Williamson nanofluid flow is better than the absence of $\alpha$. Physically, $\lambda$ is proportional to square root of kinematic viscosity $\upsilon^{*}$. Due to this the low viscosity in Williamson fluid flow generate high skin friction coefficient along $x^{*}$-direction.

Figure 11 illustrate that the $\alpha$(“Stretching Ratio Parameter”) on ${\text{Re}}_{x}^{1/2} C_{fx}$ with Williamson and nanofluid flow cases. It is clear that the skin friction coefficient monotonically declines along $x^{*}$ direction for different ascending values of $\alpha$. We decide, the Williamson liquid is more significant while comparing to nanoliquid motion. Physically, the kinematic viscosity is more in Williamson liquid motion. Due to this, the fluid produces more skin friction in surface.

The numerical results on $f^{\prime\prime}(0)$ (“Velocity Gradients”) as $\lambda = 0$ for various values of $\alpha$ in Table 1. Also, Table 2 exhibited coefficient of skin friction with different results of $\alpha$ for $\lambda = 0$. The outcomes are matched with those of Wang ^79^, Ariel et al. ^80^. It is noticed that very good agreement up to eight decimal places. Tables 3 and 4 Explored the Heat and Mass Transfer rates with various numerical numbers for $\alpha = 0$.

**Table 3:** Numerical values of ${\text{Re}}_{x}^{ - 1/2} Nu_{x}$ with different parameters of $\Gamma_{1}$, $\Gamma_{2}$, $H$ $N_{t}$ , $N_{b}$, $\Pr$ and $R_{d}$ For $\alpha = 0$.

$\Gamma_{1}$	$\Gamma_{2}$	$H$	$N_{t}$	$N_{b}$	$\Pr$	$R_{d}$	−${\text{Re}}_{x}^{ - 1/2} Nu_{x}$
0.2							0.03121
0.4							0.07577
0.6							0.11430
0.8							0.14530
	0.2						0.01051
	0.4						0.01046
	0.6						0.01043
	0.8						0.01042
		0.2					0.01054
		0.4					0.01098
		0.6					0.01131
		0.8					0.01157
			0.2				0.01054
			0.4				0.01052
			0.6				0.01049
			0.8				0.01046
				0.2			0.01058
				0.4			0.01056
				0.6			0.01053
				0.8			0.01050
					0.1		0.00974
					0.2		0.00998
					0.3		0.01038
					0.4		0.01071
						0.1	0.00801
						0.2	0.00865
						0.3	0.00929
						0.4	0.00992

**Table 4:** Numerical values of $Sh{\text{Re}}_{x}^{ - 1/2}$ with different parameters of $\Gamma_{1}$, $\Gamma_{2}$, $H$, $N_{t}$ , $N_{b}$, $Le$ $M$, and $\lambda$ For $\alpha = 0$ .

$\Gamma_{1}$	$\Gamma_{2}$	$H$	$N_{t}$	$N_{b}$	$Le$	$M$	$\lambda$	−$ShRe_{x}^{ - 1/2}$
0.3								0.00516
0.4								0.00501
0.6								0.00480
0.8								0.00466
	0.1							0.00564
	0.2							0.01515
	0.3							0.02444
	0.4							0.03267
		0.01						0.00591
		0.02						0.00590
		0.03						0.00589
		0.04						0.00588
			0.1					0.00598
			0.2					0.00578
			0.3					0.00229
			0.4					0.00541
				0.1				0.00426
				0.2				0.00519
				0.3				0.00551
				0.4				0.00568
					0.1			0.00564
					0.2			0.00578
					0.3			0.00581
					0.4			0.00604
						0.1		0.00578
						0.2		0.00578
						0.3		0.00578
						0.4		0.00578
							0.1	0.00578
							0.2	0.00578
							0.3	0.00578
							0.4	0.00578

## Conclusions

This article related to the influence of thermal radiative and heat absorption on 3D Williamson nanoliquid motion via linear stretching surface. It is analyzed numerical technique with 4th order R–K–F (“Runge–Kutta–Fehlberg”) scheme. We have noticed, the main points in present mathematical model as below:

- The velocity of Williamson nanofluid motion is high when compared to nanofluid motion with high effect of $M$.
- Skinfriction coefficient (“along $x^{*}$-axis”) is high in absents of $\alpha$(“Williamson parameter”) while comparing to presence of $\alpha$(“Williamson parameter”) with higher statistical values of $\lambda$.
- Skinfriction coefficient (“along $x^{*}$-axis”) is high in Williamson liquid motion while comparing to nanoliquid motion with higher statistical values of $\alpha$.
- The temperature is high in non-newtonian nanofluid while compared with enhance statistical values.
- The fluid velocity is higher in Williamson fluid while compared with nanofluid motion. Because of electrical conductivity.

## Data Availability

The datasets generated and/or analysed during the current study are not publicly available but are available from the corresponding author on reasonable request.

## Abbreviations

u_1_, v_1_, w_1_: Velocity components along *x**, *y**, *z**
$$(x^{*} ,y^{*} )$$: Cartesian coordinate’s
$A_{1}$: First Rivlin-Erickson tensor
$C^{*}$: Nanoparticle volume fraction
$C_{f}^{*}$: Skin friction coefficient
$$C_{p}^{*}$$: Specific heat
$$D_{n}$$: Thermal diffusivity
$$D_{B}$$: Brownian diffusion
$$D_{T}$$: Thermophoresis diffusion $$(m^{2} \,s^{ - 1} )$$
$f$: Dimensionless stream function
$$f^{^{\prime}}$$: Dimensionless velocity
$$H$$: Heat absorption $$\frac{{Q_{0} }}{{a_{1} \left( {\rho^{*} C_{p} } \right)_{f} }}$$
$k^{*}$: Mean absorption coefficient
$$Le$$: Lewis number $$\left( { = \frac{{\alpha_{m}^{*} }}{{D_{B} }}} \right)$$
$$M$$: Magnetic parameter $$= \left( {\frac{{\sigma^{*} M_{0}^{2} }}{{\rho_{f}^{*} .a_{1} }}} \right)$$
$N_{b}$: Brownian motion coefficient $= \frac{{\tau^{*} D_{B} \left( {C_{w}^{*} - C_{\infty }^{*} } \right)}}{{\alpha_{m}^{*} }}$
$$N_{t}$$: Thermophoresis parameter $$= \frac{{\tau^{*} D_{T} (T_{w}^{*} - T_{\infty }^{*} )}}{{T_{\infty }^{*} \alpha_{m}^{*} }}$$
$$Nu_{x}$$: Nusselt number
$$\Pr$$: Prandtl number $$\left( {\upsilon /\alpha_{m} } \right)$$
$q_{n}$: Surface microorganism flux
$q_{r}$: Radiative heat flux $(W\,m^{ - 2} )$
$$R_{d}$$: Radiation parameter $$= \frac{{16\sigma^{*} T_{\infty }^{3} }}{{3k^{*} \left( {\rho C_{p} } \right)_{f} }}$$
$${\text{Re}}_{x}$$: Reynolds number
$$T^{*}$$: Fluid temperature $$(K)$$
$T_{w}^{*}$: Temperature of the fluid
$T_{\infty }^{*}$: Ambient fluid Temperature

## Greek symbols

$$\theta$$: Dimensionless temperature
$$\phi$$: Dimensionless concentration
$$\lambda$$: Williamson parameter $$\Gamma x^{*} \sqrt {{\raise0.7ex\hbox{${2a_{1}^{3} }$} \!\mathord{\left/ {\vphantom {{2a_{1}^{3} } {\upsilon^{*} }}}\right.\kern-0pt} \!\lower0.7ex\hbox{${\upsilon^{*} }$}}}$$
$$\alpha_{m}^{*}$$: Thermal conductivity $$= \frac{{\mu_{0}^{*} }}{{\rho_{f}^{*} }}$$
$$\alpha$$: Stretching ratio parameter $$= {\raise0.7ex\hbox{${b_{1} }$} \!\mathord{\left/ {\vphantom {{b_{1} } {a_{1} }}}\right.\kern-0pt} \!\lower0.7ex\hbox{${a_{1} }$}}$$
$$\sigma^{*}$$: Boltzmann constant $$(wm^{ - 2} K^{ - 4} )$$
$\mu_{\infty }^{*}$: Dynamic viscosity at infinite shear rate
$$\upsilon^{*}$$: Kinematic viscosity $$\left( {\mu_{0}^{*} /\rho_{f}^{*} } \right)$$
$\tau^{*}$: Parameter defined by $\left( {\rho^{*} C_{p} } \right)\backslash \left( {\rho^{*} C_{f} } \right)$
$$\eta$$: Similarity variable
$\mu_{0}$: Limiting viscosity at zero
$\tau$: Extra stress tensor
$$\rho$$: Fluid density $$(Kg\;m^{ - 3} )$$
$$\Gamma_{1}$$: Thermal Biot Number $${\raise0.7ex\hbox{${h_{1} }$} \!\mathord{\left/ {\vphantom {{h_{1} } {k_{1}^{*} }}}\right.\kern-0pt} \!\lower0.7ex\hbox{${k_{1}^{*} }$}}$$
$$\Gamma_{2}$$: Concentration Biot Number $${\raise0.7ex\hbox{${h_{2} }$} \!\mathord{\left/ {\vphantom {{h_{2} } D}}\right.\kern-0pt} \!\lower0.7ex\hbox{$D$}}$$

## Subscripts

$\infty$: Condition at free stream
